# Therapeutic Effect of Sodium Selenite and Zinc Sulphate as Supplementary with Meglumine Antimoniate( Glucantime®) Against Cutaneous Leishmaniasis In BALB/C Mice

**Published:** 2010-09

**Authors:** F Zamani Sorkhroodi, AM Alavi Naeini, AR Zahraei Ramazani, MR Aghaye Ghazvini, M Mohebali, SA Keshavarz

**Affiliations:** 1 Dept. of Nutrition and Biochemistry, School of Health, Tehran University of Medical Science, Iran; 2 Dept. of Entomology, School of Health, Tehran University of Medical Science, Iran; 3 Isfahan Center of Public Health Training and Research, Institute of Public Health Research, Tehran University of Medical Science, Iran; 4 Dept. of Medical Parasitology and Mycology, School of Health, Tehran University of Medical Science, Iran

**Keywords:** *Leishmania major*, Sodium selenite, Zinc sulphate, Meglumine Antimoniate (glucantime*®)*, BALB/c

## Abstract

**Background:**

Successful therapy of leishmaniasis depends on effective cellular immune response. We evaluated the effectiveness of sodium selenite and zinc sulphate as known immunomodulator materials, in combination with Glucantime® in treatment of cutaneous leishmaniasis lesions resulting from *Leishmania major* in susceptible animal model.

**Methods:**

Thirty three female mice weighing 18–20 g at the age of 7–8 week infected with *L. major* were randomly divided into 3 groups: group1: treated by sodium selenite (0.35 mg/kg for 30 days), group2: treated by zinc sulphate (2 mg/kg for 30 days) and group3: treated by distilled water (0.01 ml/gr body weight for 30 days) as control. All groups received Glucantime® as a standard anti- leishmanial agent (60 mg/kg, ip) for 14 days. To assess the results of treatment measurement of lesions size and parasitological tests were done weekly.

**Results:**

The lesion sizes increased continuously in sodium selenite group.Although, in zinc group did not increase compared to baseline But with considering the time- group interaction there was no significant difference between zinc and control group during this study. There was no difference between lesion sizes and Leishmanial loads in the interventional and control groups, respectively.

**Conclusion:**

Sodium selenite and zinc sulphate at mentioned doses and duration of treatment did not show any treatment effect on cutaneous leishmaniasis caused by *L. major* in BALB/c mice. Increasing the dose of supplements and considering the follow up period after treatment can help more certain conclusion.

## Introduction

Cutaneous leishmaniasis is an infection caused by *Leishmania* protozoa, which are usually transmitted by the bite of various species of phlebotomine sand flies (1). It is endemic in 88 countries. Approximately 350 million people are thought to be at risk. Cutaneous leishmaniasis due to *L. major* (CLM) is a great health problem and prevails in rural districts of 16 out of 30 provinces in Iran (2).

One of the most effective drugs in leishmaniasis treatment, which used systematically, is pentavalent antimonies such as Glucantime® (3).

In human and experimental cutaneous leishmaniasis, development of protective immunity is dependent on the generation of INFγ-producing T cells. INFγ activates infected macrophages to eliminate the parasite via reactive oxygen and reactive nitrogen(4).Both of these intermediates reactive oxygen intermediates (ROI) and reactive nitrogen intermediates (RNI) are important in macrophages and in vitro suppression of each intermediate resulted in failure of protozoa killing in macrophages (5).

Recent studies in animal models showed that successful leishmaniasis therapy depends on effective cellular immune response. Thus immunotherapy should be used in combination with chemotherapy (6,7). Therefore, we can use immunomodulator components as valuable tools to modulate and enhance immune responses. Experimental studies showed that zinc deficiency could change immune functions from cellular Th1 responses to humoral Th2 response (8). On the other hand, zinc deficiency could be resulted some skin lesions (9,10).

Although, the other *in vitro* and *in vivo* studies have shown antileishmanial effect of zinc sulphate, this supplement in one of the human study was less effective in comparison with Glucantime® (11).

There are different reports about oxidative stress and probable effectiveness of antioxidants in leishmaniasis (12–14). Selenium is one of the important cellular antioxidants (9). There is a failure in Th1 cytokine production in parallel with selenium deficiency and supplementation with selenium in selenium deficient animals results in IL2 secretion which is secreted from Th1 (15). In addition, *in vitro* studies have shown that sodium selenite can inhibit *Leishmania donovani* growth through blocking of polyamine biosynthesis (16). Studies have shown significant decrease in serum selenium and its related enzyme glutathione peroxidase (17).

With considering few studies in sodium selenite and oral zinc effectiveness in cutaneous leishmaniasis treatment and positive effects of antioxidants (selenium and zinc) on host's immune function and in the other hand, negative effects via free radicals trapping which are important in macrophage, we decided to evaluate the effectiveness of oral sodium selenite and zinc sulphate as immunomodulator in combination with standard chemotherapy (Glucantime®)against cutaneous leishmaniasis caused by *L. major* in BALB/c.

## Materials and Methods

### Animals

Thirty-three female BALB/c mice of approximately 18–20 g weight at the age of 7–8 week were used in this experimental study. All mice were housed in climatically controlled room in plastic cages and fed with standard rodent food pellet.

### Parasites

*Leishmania major* (MRHO/IR/75/ER) Iran reference strain was obtained from the School of Public Health, Tehran University of Medical Science, Iran in NNN medium and then a sub-cultured to RPMI1640 as enriched liquid medium.

### Immunomodulator supplement and antileishmanial drug

The immunomodulator supplement was sodium selenite:( Merck, 0.35 mg/kg) and zinc sulphate: (Razi company, 2 mg/kg) and distilled water as placebo. Ampoules of meglumine antimoniate (Glucantime®) purchased from Spica, France were used 60 mg/kg (1).

### Experimental protocol

After disinfection of injection site, 0.1–0.2 ml of culture medium containing at least 2×10^6^ promastigote at stationary phase in 0.1 ml, injected at the base of BALB/c tail subcutaneously. The weight and diameter of lesions were measured before treatment. Impression smears were prepared from lesions; methanol fixed, and stained with 10% Geimsa stain in water. Almost one month after injection and appearance of lesions, treatment started. Thirty and three BALB/c mice were randomly divided into 3 groups as follows: group1: treated by sodium selenite (0.35 mg/kg for 30 days), group2: treated by zinc sulphate (2 mg/kg for 30 days) and group3: treated by distilled water (0.01 ml/gr body weight for 30 days) as control. All groups received Glucantime® as a standard anti- leishmanial agent (60 mg/kg, ip) for 14 days.

To assess the results of treatment, size of lesions measured weekly up to the end of treatment. Microscopy slides were also prepared, fixed with methanol, stained by Geimsa and examined by light microscopy (× 1000). The parasite density is graded according to grading of *Leishmania* parasites (19). Treatment efficacy was determined by comparing the diameters of skin lesions and the presence of amastigote forms between intervention and control groups.

### Statistical Analysis

The results are given as mean±SEM values. The significance of the mean difference between groups was assessed by the Student's t-test and repeated ANOVA. Parsitologic results were compared by chi- square test.

## Results

**Fig. 1:**
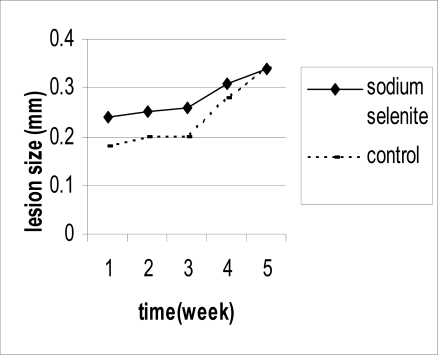
Mean of lesion sizes (mm) during treatment period in sodium selenite group compared with control group

**Fig. 2:**
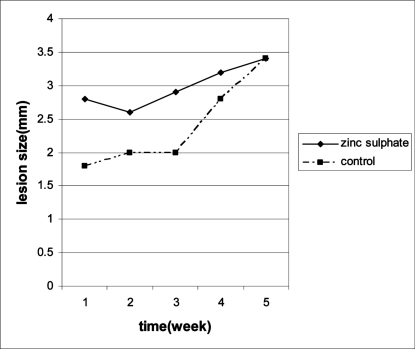
Mean of lesion sizes (mm) during treatment period in zinc sulphate group compared with control group

The mean of lesion size in sodium selenite group, zinc sulphate group and control group was 3.4±0.2 mm, 3.4±0.5 mm and 3.4±0.4 mm after 30 days post treatment, respectively. There were no significant difference between treatment groups and control group at baseline. The mean of lesion sizes increased in sodium selenite and control group compared to baseline and there were no difference between these two groups (Table 1, Fig. 1). The mean of lesion size in zinc group did not increase compared to baseline. However, with considering the time- group interaction there was no significant difference between zinc and control group (Table 1, Fig. 2).

**Table 1:** Therapeutic effect of zinc sulphate and sodium selenite on the lesion sizes (mm) of localized cutaneous leishmaniasis caused by *L. major* in BALB/c mice compared to control groups

Group	No	Lesion Size(mm)Before Intervention X^*^ ±2SD^**^	Lesion Size(mm) After Intervention X^*^ ±2SD^**^	direction	*P* value
Zinc Sulphate	11	*2.8*±0.3	*3.4*±0.5	*↑*	0.57
Sodium Selenite	11	2.4± 0.3	3.4± 0.2	*↑*	0.73
Control	11	1.8± 0.4	*3.4*±0.4	*↑*	–

* =mean

** =standard deviation

The mean lesion sizes, were not decreased significantly before (*P*=0.57) and 4 weeks (*P*=0.73) after treatment by a two-way analysis of variance (ANOVA).

After 4 weeks of treatment, there was no statistical difference in parasitological tests of lesions between treatment groups and control group (Table 2).

**Table 2:** Parsitologic tests of lesions of localized cutaneous leishmaniasis caused by *L. major* in BALB/c mice compared to control groups

Group^1^	without lesion	**0**	1+	2+	3+	4+ & more	*P* value
Sodium Selenite(n=11)	0	1	1	3	2	4	NS^2^
Zinc Sulphate (n=11)	0	1	1	3	2	4	*P*=0.16
Control (n=11)	2	1	1	5	1	1	

1 Grading of *Leishmania* parasites

2 Not significant

1+: 1–10 parasite/1000 fields 2+:1–10 parasites/100 fields 3+: 1–10 parasites/10 fields 4+ & more: more than 10 parasites/10 fields

## Discussion

Leishmaniasis is a worldwide parasitic disease currently treated with expensive compounds and severe side effects, and are frequently ineffective, emphasizing the importance to search new compounds against this disease. The standard agents for leishmaniasis such as pentavalent antimonials, pentamidine, and amphotericin B and miltefosine have the disadvantages of repeated parenteral injection and of toxicity (18,19).

All groups received Glucantime® but the mean of lesion size increased during the treatment period and there was no significant difference between zinc and control group and also between selenite and control group. Maybe high sensitivity of this animal model against *L. major* (20) is the cause of this progressive process, although sodium selenite has antileishmanial activity against *L. major* promastigotes *in vitro* (16). In the present investigation, sodium selenite had no activity at the dose of 0.35 mg/kg *in vivo* and there were no difference between treatment and control group in parasitological tests. This dose had low toxicity in mice (21). Maybe the use of higher doses had different effect in cutaneous leishmaniasis treatment. Moreover, we used WHO grading of *Leishmania* parasites for lesion. Maybe this grading cannot show the exact difference between groups (22).

Reactive oxygen intermediates (ROI) and reactive nitrogen intermediates (RNI) are responsible for parasite killing in macrophages and suppression of each radicals inhibits macrophage activity (5). In Bisti study, iron injection as oxidant in inhibited the parasite growth and progress in lesion size (23) which indicates the role of free radicals in leishmaniasis treatment. Maybe the antioxidant role of selenium (9) is the cause of this result.

Three studies showed the effectiveness of zinc sulphate injection in treatment of cutaneous leishmaniasis (24–26). Nevertheless, oral zinc had different effects. Zinc sulphate was effective in treatment of cutaneous leishmaniasis at the dose of 10 mg/kg for 5 days in mice (27). These results indicate that high doses in short time is more effective than low dose in long time. This study used MHOM/IQ/93/MRC6 strain that is different from our parasite strain. Maybe our different result is due to this issue.

In another study (28), zinc sulphate was effective in treatment of cutaneous leishmaniasis at the dose of 2 mg/kg for 45 days in human. The treatment time and zinc dose is similar to our study but the probable cause of different result is the sensitivity to *L. major* causes cutaneous leishmaniasis in human but it can cause both visceral and cutaneous leishmaniasis in Balb/c (20). The dose of 2 mg/kg can stimulate immune system of mice (29). Higher doses have genotoxic effect on mice (30). Oral zinc did not have any effect on acute cutaneous leishmaniasis (11). In 60 patients with cutaneous leishmaniasis and the control group of 100 healthy volunteers from the same area, levels of serum Zn were significantly lower than the control group (*P*<0.001) (31). In Faryadi et al. study there was also similar results (32), in which the acute and chronic cutaneous leishmaniasis patients had significantly lower Zn level as compared to the control subjects (32).

Two recent studies have suggested the use of blood zinc concentration as a means for estimating the prognosis of CL (31,32). In our study, oral zinc sulphate as a supplement was mostly effective compared to the baseline in treatment of cutaneous leishmaniasis.

The cause of acute cutaneous leishmaniasis is *L. tropica*, which is different from *L. major*. *In vitro* studies showed dose dependent anti-*Leishmania* effect of zinc sulphate (30). Maybe higher dose had effectiveness on treatment although it has genotoxic effect.

In conclusion, results of the present investigation indicate that sodium selenite at the dose of 0.35 mg/kg and zinc sulphate at the dose of 2 mg/kg does not have any effect on cutaneous leishmaniasis treatment in animal model. Use of higher doses and considering a follow up period may help the certain conclusion.
